# A Surgically Treated Case of Ureterovesical Amyloidosis of the Bladder in a Patient with Idiopathic Thrombocytopenia

**DOI:** 10.1155/2018/1059349

**Published:** 2018-09-06

**Authors:** Sung Han Kim, Weon Seo Park, Jae Young Joung, Kang Hyun Lee, Jinsoo Chung, Ho Kyung Seo

**Affiliations:** ^1^Department of Urology, Center for Prostate Cancer, National Cancer Center, Goyang, Republic of Korea; ^2^Department of Pathology, Center for Prostate Cancer, National Cancer Center, Goyang, Republic of Korea

## Abstract

Idiopathic thrombocytopenia (ITP) is a bleeding disorder involving the destruction of platelets by the immune system. Systemic amyloidosis is another bleeding disorder involving amyloid deposits that create defects in coagulation and increased prothrombin and thrombin times. We report a 52-year-old man with ITP and new two-month-duration, painless gross hematuria without clot formation resulting in amyloidosis involving the ureterovesical area of the bladder. He had osteopenia, hypertension, and moderate thrombocytopenia due to ITP diagnosed 7 years previously. Cystoscopic examination with urine cytology and computed tomography imaging detected a 2-cm protruding solid bladder mass involving the left ureteral orifice and trigone and left mild hydroureteronephrosis, suggesting bladder cancer. Transurethral resection of the bladder mass was performed to confirm amyloidosis involvement in the ureterovesical junction of the bladder and ureter. Four weeks postoperatively, intermittent gross hematuria remained; hence, left ureteroneocystostomy was performed. Regular follow-up showed no signs of hematuria or intravesical recurrences for 14 months.

## 1. Introduction

Immune thrombocytopenia (ITP), previously called idiopathic thrombocytopenia purpura, results from systemic antibody-mediated platelet destruction combined with impaired platelet production and comprises a syndrome of diverse immune-mediated disorders with differing pathogeneses, natural histories, and responses to therapy [1]. Treatment may be aimed at immediate and rapid control of life-threatening hemorrhaging or reducing mucosal bleeding symptoms. Fortunately, life-threatening or severe bleeding is a rare event, with up to 9.6% of adults experiencing major hemorrhaging [1]. A standard goal of therapy of ITP is to increase the platelet count to prevent subsequent hemorrhaging.

Amyloidosis is another systemic disease affecting many organs, including the genitourinary tract that is characterized by the deposition and accumulation of hyaline, eosinophilic, and proteinaceous materials in various tissues. Localized amyloidosis of the genitourinary tract is a rare entity characterized by small pseudotumors localized in the renal pelvis, ureter, or bladder [2]. The clinical and radiologic features mimic urinary tract cancer, and local surgical treatment is indicated. In most cases, the prognosis is excellent; however, disease recurrence is possible.

Very few case reports of concomitant ITP and amyloidosis in diverse organs such as the lung [3] and skin [4] exist. We report a case of a 52-year-old man with ITP and amyloidosis of the bladder. The patient had underlying ITP presenting with recurrent two-month-duration gross hematuria and mild hydronephroureterosis treated with transurethral resection of the bladder amyloidosis and subsequent ureteroneocystostomy of the ureter amyloidosis.

## 2. Case Report

A 52-year-old man presented with intermittent, two-month-duration painless gross hematuria without clot formation and was referred to the Urology Department for evaluation of urologic malignancy. He had multiple bruises and a history of nasal bleeding during childhood. Low platelet levels (34000-38000/*μ*L) and high lactate dehydrogenase titer (244-278 U/L) were found during every blood test he had undergone; however, no further specific examination of thrombocytopenia was performed because he refused to visit a tertiary hospital until ITP and severe unstoppable nasal bleeding were diagnosed at age 45 years. In addition to thrombocytopenia with serum platelets less than 20,000 mg/dL, the patient had underlying diseases such as osteopenia and hypertension without any operative history or family history. The renal function was within the normal range including serum creatinine less than 0.9mg/dL and estimated glomerular filtration rate greater than 90 mL/min/1.73m^2^.

**Figure 1:**
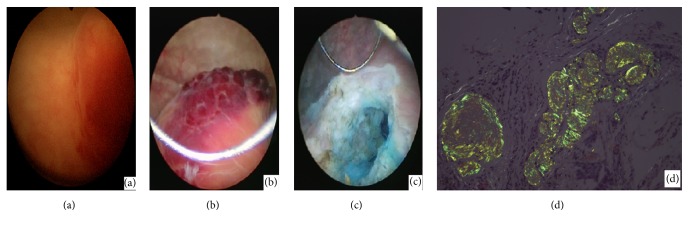
(a) Preoperative and (b, c) intraoperative cystoscopic findings of abnormal bladder lesions. (d) Deposits of amyloid material showing birefringence under polarized light (Congo red stain) (×200).

**Figure 2:**
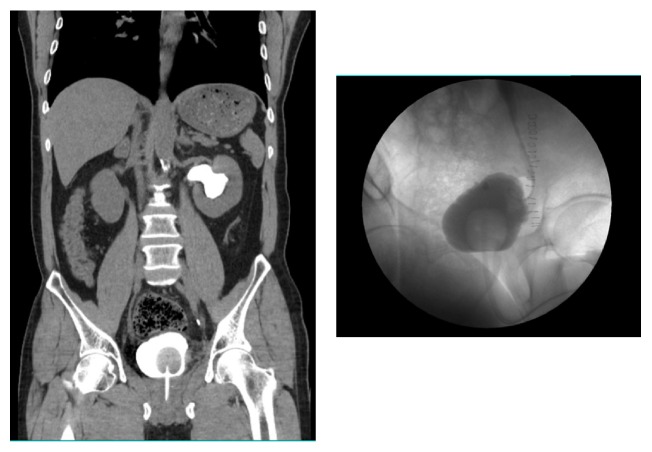
Computed tomography showing (a) abnormal elevation of the left ureterovesical area of the bladder with hydronephroureterosis and (b) degree of hydronephroureterosis with kinking ureter.

After cystoscopic examination with urine cytology and computed tomography (CT) imaging for the evaluation of urologic malignancy, a 2-cm protruding solid bladder mass involving the left ureteral orifice and trigone of the bladder with left mild hydroureteronephrosis were detected, suggesting bladder cancer (Figures 1(a) and 2). Transurethral resection of the bladder mass was performed to confirm pathologic amyloidosis involvement of the left ureteral orifice of the bladder after transfusion of five packs of platelet-concentrated plasma and three packs of fresh-frozen plasma (Figures 1(b) and 1(c)). Pathologically amorphous nodular materials were noted in the subepithelial connective tissue and smooth muscle layers. Deposits were noted in resected bladder tissues, including the left ureteral orifice, without any involvement of the periureteral tissues. Mild stromal fibrosis was also seen. Congo red-stained sections of the materials were light green under polarized light. Further immunohistochemistry results were positive for kappa but not for lambda (Figure 1(d)).

**Figure 3:**
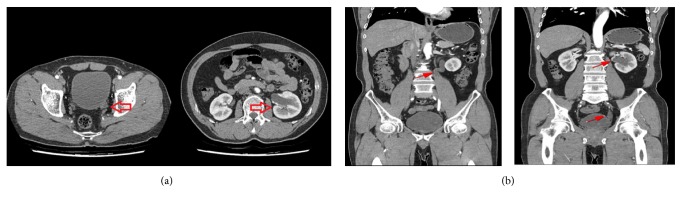
Postoperative follow-up imaging of cystographic computed tomography showing no leakage at anastomosis of ureteroneocystostomy with ureteral stent in situ.

At 2 weeks postoperatively, bone marrow biopsy confirmed polyclonal T-cell receptor gene rearrangement during multiplex polymerase chain reaction and oligoclonal immunoglobin H-chain gene rearrangement during seminested PCR. Four weeks after transurethral resection, the patient still experienced intermittent gross hematuria and left hydronephroureterosis on CT urography. Further left ureteroneocystostomy with excision of the ureterovesical area of the bladder was performed. We made an oblique cystotomy under lower midline incision and dissect the intramural ureter with mass. The mass was delivered through the incision and resected with safety margin from the ureter. The original ureteral orifice was closed. The remaining ureter was reintroduced into the bladder and made neoureteral orifice after submucosal tunneling. The cystotomy site was closed with a standard two-layer manner. Several packs of platelet-concentrated plasmas were infused intraoperatively. A clear resection margin was finally confirmed by pathology. 7 days postoperative, CT cystography confirmed the healed anastomotic site between bladder and ureter (Figure 3) and the patient was discharged. The internal ureteral stenting placed in the ureter was removed under cystoscopy on postoperative 10^th^ day at the outpatient clinics. No further hematuria and hydronephroureterosis were observed during 14-month follow-up.

## 3. Discussion

Over the past decade, the diagnosis and treatment of autoimmune disorder ITP, which is characterized by a severe reduction in the peripheral blood platelet count (less than 100 × 10^9^/L), have been expanded [1, 5]. Adult ITP occurs in approximately 2 to 4 out of 100,000 individuals, and treatment may be aimed at increasing the platelet count, preventing bleeding, inducing remission, controlling life-threatening hemorrhaging immediately and rapidly, and controlling or reducing mucosal bleeding symptoms; bleeding risks, specifically hemorrhaging and intracranial hemorrhaging, represent the most serious complications for patients with ITP [1].

Our patient also underwent two surgical resections of the bladder mass because of intermittently continuous hematuria and blood transfusion. Although this case was not treated with systemic steroid therapy after surgery because the bleeding had completely disappeared, relapse is common and occurs in approximately 50% within 6 months; an additional 25% experience relapse beyond 1 year, even after corticosteroid treatment [6]. When relapse occurs due to failed corticosteroid therapy, intravenous immune globulin G or anti-Rh(D) is used as an upfront therapy to stop bleeding and increase the platelet count to maintain the desirable level of ≥100 × 10^9^/L [7]. Standard second-line options for maintaining platelets and further inducing remission include splenectomy, rituximab, and thrombopoietin receptor agonists such as romiplostim and eltrombopag.

Amyloidosis is a protein disorder characterized by the extracellular deposition of fibrillar proteins in solid organs or tissues. Its prevalence is evenly distributed during the fifth, sixth, and seventh decades of life for men and during the sixth decade of life for women [8]. The mechanism of amyloidosis of the urinary tract is still unknown; however, abnormal protein metabolism and chronic urinary tract infections or repeated inflammation of the mucosa or submucosa are the best pathogenetic theories [9]. Amyloidosis can present as a very rare primary form associated with nonmalignant processes or as a secondary form with chronic inflammatory processes [2, 8, 9]. However, in this case, ITP was a predisposing disease primarily affecting the bladder.

Intravesical amyloidosis can mimic bladder cancer, as in this case, with intermittent, two-month-duration, painless gross hematuria, lower urinary tract symptoms, or a mass on imaging. The definitive diagnosis depends largely on histopathology confirmed by positive Congo red staining. Pelvic CT and cystoscopy did not provide any diagnostic information; however, uneven thickening of the bladder wall caused suspicion of a neoplastic lesion, resulting in surgical excision including transurethral resection and ureterectomy with ureteroneocystostomy. Postoperative recovery is good and close follow-up is recommended after discharge because of the possibility of malignancy, with an estimated recurrence rate of up to 54% [10]. Adjuvant intravesical instillation of dimethyl sulfoxide and oral colchicine therapy have been performed with promising results.

Few case reports have reported amyloidosis with underlying ITP [3, 4]. No pathogenetic mechanism has been proven or suggested regarding the combined occurrence of two diseases; however, both diseases are hematologic immune-related disorders associated with chronic inflammation. Further genomic analyses are recommended to obtain further pathogenetic information. And there is no consensus how to treat localized amyloidosis especially ureter amyloidosis with ITP. This case shows surgical approach using ureter reimplantation can be a better option than radiation or observation in this case of amyloidosis. Further information about this case is that immune thrombocytopenia can be related to localized amyloidosis with a predisposing factor.
